# Eggs of the copepod *Acartia tonsa* Dana require hypoxic conditions to tolerate prolonged embryonic development arrest

**DOI:** 10.1186/s12898-018-0217-5

**Published:** 2019-01-15

**Authors:** Tue Sparholt Jørgensen, Per Meyer Jepsen, H. Cecilie B. Petersen, Dennis Steven Friis, Benni Winding Hansen

**Affiliations:** 1 Department of Science and Environment, Roskilde University, Roskilde, Denmark; 2 Department of Environmental Science-Environmental Microbiology & Biotechnology, Aarhus University, Roskilde, Denmark

**Keywords:** Resting eggs, Quiescence, Copepod, *Acartia*, Calanoida, Embryonic arrest

## Abstract

**Background:**

Copepods make up the largest zooplankton biomass in coastal areas and estuaries and are pivotal for the normal development of fish larva of countless species. During spring in neritic boreal waters, the copepod pelagic biomass increases rapidly from near absence during winter. In the calanoid species *Acartia tonsa*, a small fraction of eggs are dormant regardless of external conditions and this has been hypothesized to be crucial for sediment egg banks and for the rapid biomass increase during spring. Other eggs can enter a state of induced arrest called quiescence when external conditions are unfavourable. While temperature is known to be a pivotal factor in the transition from developing to resting eggs and back, the role of pH and free Oxygen in embryo development has not been systematically investigated.

**Results:**

Here, we show in a laboratory setting that hypoxic conditions are necessary for resting eggs to maintain a near-intact rate of survival after several months of induced resting. We further investigate the influence of pH that is realistic for natural sediments on the viability of resting eggs and document the effect that eggs have on the pH of the surrounding environment. We find that resting eggs acidify their immediate surroundings and are able to survive in a wide range of pH.

**Conclusions:**

This is the first study to demonstrate the importance of hypoxia on the survival capabilities of *A. tonsa* resting eggs in a controlled laboratory setting, and the first to show that the large majority of quiescent eggs are able to tolerate prolonged resting. These findings have large implications for the understanding of the recruitment of copepods from sediment egg banks, which are considered the primary contributor of nauplii seeded to pelagic populations in nearshore habitats in late spring.

**Electronic supplementary material:**

The online version of this article (10.1186/s12898-018-0217-5) contains supplementary material, which is available to authorized users.

## Background

Adversity is normal in the life of many organisms. Zooplankton often experience periods of unfavourable conditions such as salinity changes, temperature changes, scarcity of food items and/or high predation pressure. Because of this, a variety of coping strategies are being used to maintain a population when external conditions are not favourable. For the group Copepoda, these strategies include migration, morphological changes, and resting developmental stages [1–3]. In this paper, we address the physiological aspect of an adversity coping strategy of an important coastal species using laboratory reared copepods. Copepods are one of the most abundant groups of multicellular animals on earth, as regards both their biomass and species richness [4]. They have a major influence on aquatic ecosystems, including the flow of organic Carbon in pelagic waters towards the ocean floors [5, 6] and the numerous food webs sustained [7] or parasitized [8] by copepod populations. In a number of coastal areas, copepods constitute the largest biomass of zooplankton [9, 10] and are a main food source for fish larvae [11]. The calanoid copepod *Acartia tonsa* Dana, 1849 is one of the most abundant copepod species in boreal coastal waters and estuaries, and is an important model species in ecophysiology [12–19]. The species has been found globally and has developed strategies of survival to adapt to local conditions. For example, only some *A. tonsa* populations produce resting eggs [20]. The life cycle of copepods consists of an embryonic stage followed by a nauplius larva and copepodite stage before the adulthood is reached [21]. *A. tonsa* eggs are spawned by the female copepod directly into the water column. In shallow water habitats, these eggs quickly sink to the seabed, where they can be buried and exposed to hypoxia, depending on external conditions. *A. tonsa* eggs are spherical and have a polymorphic surface ornamentation of spines which has been hypothesized to be important for performing a range of functions such as predation avoidance [22–24]. Depending on the temperature of the water, *A. tonsa* embryogenesis can take between 8 and 48 h, after which a nauplius larva will emerge from the egg [25, 26]. The rate at which the naupliar and copepodite stages progress are similarly dependent on the water temperature. This rate of progression is also dictated by nutrition. At 17 °C with sufficient nutrition, animals reach the adult stage in 2 weeks and adult females can remain reproductively active for 3–4 weeks, producing up to 50 eggs per day depending on the availability of food [14, 27]. Eggs from at least 42 copepod species, including *A. tonsa*, have the ability to enter a state of developmental arrest called quiescence if conditions are unfavourable [28]. One well-documented environmental cue that forces eggs of *A. tonsa* into quiescence is low temperatures, which occur during the winter when also food items such as microalgae are scarce [29, 30]. A small fraction of newly produced eggs is unable to undergo embryogenesis immediately [31]. This state is termed embryonic diapause. Diapaused eggs are thought to transition into quiescence after a refractory time that can range from days to months [26, 32]. In this study, we use the term embryonic arrest which covers any delayed hatching as all current methods are unable to differentiate between types of eggs noninvasively if at all. Embryonic arrest has been shown to have no effect on offspring, which are able to progress through their life history [30]. Readers are referred to two recent papers for more thorough review of diapause in marine copepods [3, 28].

The life history of *A. tonsa* depends on geographical location. For example, populations in warm water can remain present over the cause of a year while the biomass of *A. tonsa* in Boreal coastal waters exhibit large seasonal fluctuations [33]. Here, the species is almost absent in the water column during winter, after which the pelagic biomass increases in the spring and reaches a maximum in the summer [34]. During the late autumn months, the pelagic population of *A. tonsa* disappears. The autumn population produces an ‘egg-bank’ in and on the sediment, which explains the appearance of pelagic copepods when conditions are favorable after the winter [34–36]. The sediment environment consists of a mixture of sand, silt, and clay, mixed with decomposing organic material. The properties of the sediment can vary significantly and factors such as low pH, Oxygen levels, and sulphide levels can influence the survival of copepod eggs [35, 37]. Predation can also reduce recruitment from copepod resting eggs in or on the sediment [38]. Importantly, resting eggs depend on external factors, such as storms, to be buried in the sediment and re-emerge from the sediment in order to be exposed to favourable conditions and successfully complete their life cycle. The precise mechanisms that regulate the exit from embryonic arrest have not been exhaustively explained. Here, we propose that a necessary cue for exiting quiescence and entering into embryogenesis is Oxygen availability, and present evidence that hypoxia is necessary for arresting the embryogenesis and for the survival of eggs in long term arrest. Furthermore, we explore the tolerance to low pH values of resting eggs and find that the effect of even a realistically acidic environment does not affect the viability of resting eggs. In the present study, we have mimicked benthic sediment conditions such as temperature and pH to gain a broader understanding of the mechanisms required for embryonic arrest and the successful resumption of embryological development.

## Results

We have attempted to set up an experimental system where the impact of abiotic factors on *A. tonsa* resting eggs can be investigated. This approach differs from many previous efforts, where more environmentally realistic setups have been favoured [29, 35, 39]. We believe that our approach has the great advantage of knowing the exact age of embryos, allowing studies of their fate that would not be possible with a more natural setup e.g. using sediment cores where the exact age of eggs is unknown. The effect of Oxygen availability and pH are investigated in this study, as these are considered to fluctuate and to be biologically important in the benthic zone throughout the year. In these experiments, a setup in closed containers was preferred over an open system for simplicity and to completely separate the replicates. The effect of sulphide on eggs has previously been thoroughly investigated, which is why this factor is not considered in the present study [37].

1. Oxygen permeability of sample containers.

**Fig. 1:**
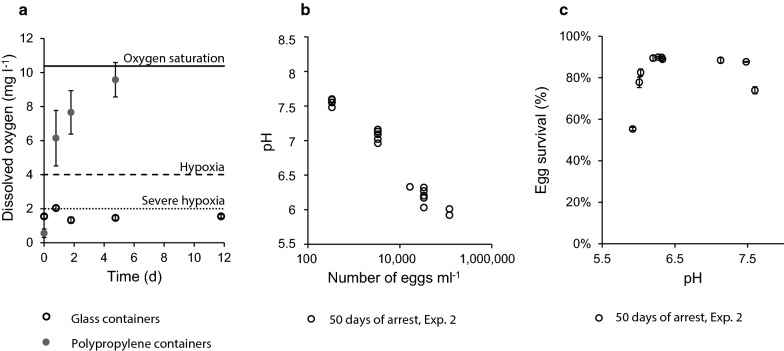
Oxygen permeability, effect of egg abundance on pH, and effect of pH on viability of eggs. **a** Oxygen permeability in experimental containers. Data points are means of 5–20 replicates and error bars are standard deviations. Oxygen levels in glass containers remain low and stable over several days whereas the Oxygen concentration in polypropylene containers increases, reaching near saturation after less than 5 days. Lines represent Oxygen saturation (10.4 mg O_2_ L^−1^), hypoxia (4 mg O_2_ L^−1^), and severe hypoxia (2 mg O_2_ L^−1^). **b** Samples with increasing egg abundance have decreasing pH. Data points are individual samples. Eggs were counted at the start of the experiment. In Exp. 2, where eggs were arrested for 50 days in abundances of 300–120,000 mL^−1^, each increase in egg abundance resulted in a drop in pH. Thus, the samples with lowest egg abundance have a pH of 7.5 while the samples with highest egg abundance have a pH of ≤ 6. The initial pH of 8 in control samples did not change during the duration of the experiment (data not shown). **c** The survival rate of eggs is stable over a wide range of pH. Ten samples from Exp. 2 were hatched in triplicate and the emerging nauplii counted. Error bars are s.d. For samples with pH 6.0–7.5, the survival is stable and between 78 and 90% ZTM. The survival rate of eggs in samples with sediment realistic acidity levels suggests that the acidity of the environment is not of great importance to egg survival. At the lowest pH in the experiment, the survival rate is 55%

In several experimental setups of eggs stored in polypropylene tubes, we observed that fewer eggs were present at each consecutive time point. This led to the hypothesis that the Oxygen permeability of the sample containers decreased survival rates of resting eggs. The possible mechanisms for the decreased survival include oxidative stress of the eggs and triggering of continuation of embryogenesis. In order to determine the oxygen permeability of the containers used in this study, the Oxygen content of 72 containers filled with initially hypoxic seawater was measured over the course of several days (Table 1 Exp. 1). The dissolved Oxygen (DO) content of 0.2 mL polypropylene containers, while almost anoxic (< 1 mg/L DO) at the beginning of the experiment, had an average of 6 mg/L DO after only 19 h (Fig. 1a, closed grey circles). The next day, samples in 0.2 mL polypropylene containers had an average of almost 8 mg/L DO. After 5 days, samples in polypropylene containers had an average of almost 10 mg/L DO, very close to the level of Oxygen solubility at 5 °C in 32 ppt seawater (Fig. 1a black line, 10.4 mg/L DO, https://water.usgs.gov/software/DOTABLES/). By contrast, and as expected, samples in air tight glass containers did not show any increase or decrease in Oxygen concentration and remained in severe hypoxia (Fig. 1a, dotted line) even after 12 days (Fig. 1a, open black circles). To confirm the negligible Oxygen exchange rate in glass containers, the Oxygen saturation of all samples with eggs in hypoxic conditions (Exp. 3 and 4, n = 159) was measured and in all cases showed hypoxic, severe hypoxic, or anoxic conditions (data not shown). The fact that polypropylene containers have a fast Oxygen diffusion rate and glass containers have a negligible Oxygen diffusion rate means that together they present a practical method for studying the effect of Oxygen availability on the rate of survival for copepod resting eggs: Polypropylene tubes will have a constant high level of dissolved Oxygen even if Oxygen is being consumed and glass containers can maintain a constant, low Oxygen level.

**Table 1:** Overview of experiments

Exp.	Test	Replicates per timepoint	Total replicates	Measurements	Variable(s)	Figures
1	Oxygen permeability of sample containers	5–20	72	O_2_	Container type: glass or polypropylene	1a
2	Egg abundance effect on pH	2–3	21	Eggs, nauplii, pH	Egg abundance	1b, c
3	Oxygen effect on resting egg	9–10	79	Eggs and nauplii, O_2_, pH	Oxygen availability: hypoxic condition	2a, b
4	Oxygen effect on resting egg	10	80	Eggs and nauplii, O_2_, pH	Oxygen availability: hypoxic condition	2a, b
5	Oxygen effect on resting egg	6–8	30	Eggs and nauplii	Oxygen availability: normoxic condition	2a, b

2. Resting eggs contribute to acidification of their surroundings.

Egg respiration by-products, microbial activity, and biogeochemical processes can acidify the benthic environment in which resting eggs of *A. tonsa* are found. Because of this, we wanted to investigate to what extent the resting eggs of *A. tonsa* themselves play a role in acidification and whether the survival rate of resting eggs is negatively affected by a low pH.

In Fig. 1b, the pH of each sample at the endpoints of Exp. 2 is plotted against the abundance of eggs at the start of the experiments. A link between an increase in the number of eggs per ml and a decrease in pH can be seen as samples with 300 eggs mL^−1^ have a pH of 7.5, whereas samples with 112,000 eggs mL^−1^ have a pH of 6.0 (Fig. 1b, initial sample pH 8.0 ± 0.1 s.d.). The sample with the lowest pH observed (pH 5.9) and the highest egg abundances are equivalent to the estimated pH inside the eggs [37]. The pH in control samples without eggs remained stable at 8.0 ± 0.1 s.d. after 50 days (Additional file 1, Exp. 2).

In order to determine the effect of pH on the viability of resting eggs, eggs in three subsamples from each of ten samples from Exp. 2 were hatched and the nauplii and unhatched eggs were counted in order to determine the rate of survival. While the pH varied as much as 1.5 units between the samples, the survival rate after 50 days of arrest was stable with about 90% hatching over a pH range from 6 to 7.5 (Fig. 1c). A notable difference, however, was seen at the lowest pH (and highest egg abundance of 120,000 eggs mL^−1^), where only 55% ± 0.8 s.d. of the eggs were found to hatch. These results suggest that while resting eggs affect the pH of their surroundings, they are able to withstand a wide range of pH and remain viable [40]. Accordingly, these eggs can endure embryonic arrest at abundances of at least 33,000 eggs mL^−1^ with little to no mortality caused by a low pH. This egg abundance is much higher than what has previously been reported in the sediment [35].

3. The influence of Oxygen on egg survival.

**Fig. 2:**
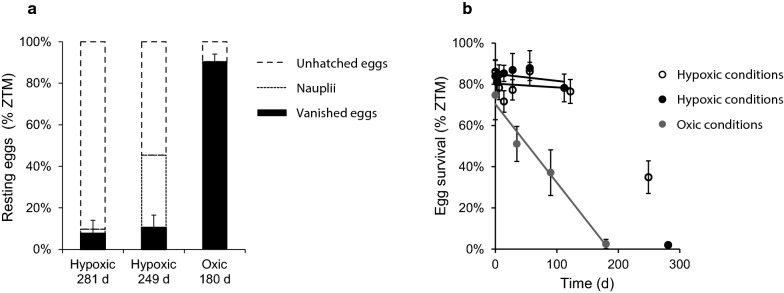
Egg loss and egg survival rate during arrest. **a** Egg loss after 6–9 months of arrest. When Oxygen is not available, egg loss is negligible, even after > 250 days of arrest. Conversely, 90% of eggs are lost after only 180 days in normoxic conditions (black bars, ZTM loss). Egg loss is presented as means of 6–10 replicates and error bars are 95% CL. Outlines of mean nauplii and unhatched egg rates are seen as dotted and dashed lines, respectively. **b** Egg survival rate in hypoxic conditions remains unchanged over 120 days but is severely reduced at ca 280 days of embryonic arrest. The survival rate (ZTM) in the two experimental setups without Oxygen were not statistically different from each other, whereas the survival rate (ZTM) for eggs exposed to Oxygen was significantly lower (p < 0.01, Fs = 5.91, d.f. (a1 + b2 − 4) = 112 & Fs = 6.80, d.f. (a1 + b2 − 4) = 113). Extinction for samples in hypoxia is reached after ca 300 days. In normoxic conditions, the mean egg survival rate (ZTM) drops between each time point, reaching near extinction after < 200 days of embryonic arrest. Data points are ZTM of 6–10 replicates, lines represent linear regressions, and error bars are 95% CL

In several experimental setups in normoxic conditions (crystallized in Fig. 1a), we observed that fewer eggs were present at each consecutive time point, potentially caused by a combination of hatching eggs and mortality [26]. Egg loss is defined here as the difference between the number of newly deposited eggs in a sample at the start of the experiment and the observed number of eggs and nauplii at the sampling time after incubation at optimal hatching conditions. Because of the invasiveness of counting eggs, we used the mean number of eggs in a sample at the start of the experiment (zero time mean number of eggs, ZTM) to calculate egg loss. In Fig. 2a, egg loss in hypoxic and normoxic conditions is visualized in samples from time points over 6 months (Exp. 3–5). Only ca 10% of eggs are lost after 9 months in hypoxia, whereas ca 90% of eggs stored at normoxic conditions are lost after 6 months (Fig. 2a, black columns). The number of hatched and unhatched eggs remains high only in samples in hypoxia (Fig. 2a, dashed and dotted columns, respectively), whereas a substantial fraction of eggs vanish in normoxic conditions. Two realistic scenarios could lead to the observed loss of eggs in normoxic conditions: either the eggs hatch, die, and degrade, or the eggs burst without re-entering embryogenesis. We conclude from these experiments that egg loss in prolonged embryonic arrest is negligible only in hypoxic conditions, and that egg loss is the main factor in the reduced survival of eggs in embryonic arrest in normoxic conditions.

In order to investigate the survival rate of resting *A. tonsa* eggs in normoxic and hypoxic conditions, we counted the number of nauplii which emerged after incubating resting eggs at optimal conditions (Table 1, Exp. 3–5). Survival rates were determined by relating the number of nauplii to the mean number of eggs in a sample at time zero (ZTM) to account for egg loss, something which several previous studies did not specify. In Fig. 2b, mean survival rates of the six to 10 replicates for each time point is presented. For all samples in hypoxic conditions, the survival rate remains above 70% of ZTM in all time points up to 122 days of arrest, but drops to ca 35% after 249 days of arrest and further to < 5% after 281 days of arrest (Fig. 2b, closed and open circles). Accordingly, the survival rate of eggs in hypoxic conditions remained largely unchanged for several months of arrest in hypoxia at 5 °C. The slight increase in survival rates seen after 56 days in both experiments in hypoxic conditions could potentially be explained by diapaused or delayed hatching eggs (sensu [20]), eggs that are unable to hatch immediately after being laid but which will enter quiescence or development after several days [27, 41].

In the experiment where resting eggs were exposed to Oxygen, the mean rate of survival for the eggs decrease for each time point relative to the mean number of eggs in each sample at time zero (ZTM, Fig. 2b, grey circles). The rate of survival of resting eggs under normoxic conditions therefore decreases as a function of time, consistent with the egg loss after 6 months seen in Fig. 2a.

The eggs that were not exposed to oxygen did not fulfil the assumptions for a linear regression since the slopes were not statistically significant different from 0 (− 0.03x + 85.06, p = 0.32 and − 0.02x + 80.31, p = 0.56). The slope for the eggs exposed to oxygen did fulfil the assumptions for a linear regression (− 0.38x + 69.71, p < 0.0001). Nevertheless, we choose to statistically compare the three slopes with a F-test as if the slopes were linear regressions. By linear regression over data points < 200 days, survival rates under normoxic and hypoxic conditions can be compared. The survival rate of eggs in samples not exposed to Oxygen were not statistically different from each other (Exp. 3 and 4), whereas the eggs exposed to Oxygen (Exp. 5) had a significantly lower survival rate than the eggs in either of the experiments did where they were not exposed to Oxygen (p < 0.01, Fs = 7.08, d.f. (a1 + b2 − 4) = 94 & Fs = 7.81, d.f.(a1 + b2 − 4) = 95). Linear regression of ZTM survival rates was chosen to reflect the negligible mortality of eggs during early months in hypoxia and to account for suboptimal sampling intervals. We believe that these results are an important addition to the knowledge about *A. tonsa* resting eggs in natural environments, as they document the importance of hypoxia for the fate of resting eggs. The preservation of eggs to cope with warm or cold periods is considered an adaptive trait of vital significance for copepods in fluctuating environments as discussed in a recent contribution by Holm et al. [28].

## Discussion

The importance of Oxygen to calanoid copepod embryo development is highlighted in a 1975 study [42], in which eggs in deoxygenated water do not hatch, even when the temperature is favourable. The survival of resting eggs under these conditions was reduced to less than a week which could be attributed to the metabolism required to maintain embryonic arrest in elevated temperatures.

The present study used closed sample containers. This could have led to the accumulation of additional substances within the sample containers such as ammonia and sulphide. A recent paper explored the link between *A. tonsa* resting egg mortality and sulphide concentration [37]. They found a correlation between elevated sulphide concentrations and resting egg mortality, but only for very high sulphide concentrations. We expect the concentration of sulphide in the sample containers to be negligible for the long term survival experiments 3–5 (Table 1). This assumption is supported by the findings in Fig. 1b and c, where the viability of eggs is constant for samples with much higher egg abundances than in the long term experiment. In dense static *A. tonsa* cultures, the level of ammonia can reach toxic limits due to the pH dependent equilibrium between NH_4_/NH_3_ [43]. The release of NH_4_/NH_3_ from *A. tonsa* eggs is unknown, but expected to be negligible. NH_3_ is more toxic than NH_4_ for marine copepods but in egg storage vials with pH below 8, the fraction of NH_3_ is less than 3% of total NH_4_/NH_3_ and unlikely to reach toxic levels. Therefore, we do not expect the accumulation of additional substances to fundamentally affect the results or conclusions of this study. For this study, a laboratory culture of *A. tonsa* was used, which may raise the concern that the findings are culture specific and do not apply to wild populations. While existing genetic resources from the culture and wild populations are currently insufficient to compare them, the behaviour of animals in the culture used and wild populations has been scrutinized. In studies comparing wild and cultured *Acartia* animals, authors have demonstrated that the behaviour, fecundity, hatching rate, and nauplii mortality is not different between wild and cultured animals [40, 44].

In experiment 2, where the effect of the egg abundance in a sample is related to the subsequent pH, we found a relationship between high egg abundances and a low pH (Fig. 1b). These results indicate that eggs contribute profoundly to the acidification of their immediate environment, potentially as a result of respiration by-products, microbial degradation of dead embryos, or the release of acidic egg content to the surroundings. It should be noted that many other factors than copepod eggs influence the sediment pH in the environment. It is interesting, however, that eggs were able to survive arrest at pH > 6.0 without large loss (Fig. 1c), indicating that eggs are not affected by sediment realistic acidity. Further, it would be interesting to investigate if the low pH of eggs is evenly distributed or concentrated in certain cells, layers, or compartments and to document the pH tolerance of nauplii, copepodites, and adult *A. tonsa* animals to explore the resilience of the different life stages. Previously, it has been reported as a personal comment that eggs can hatch at pH 6, but that nauplii die after exposure to the acidic water column [40], which seemingly contradicts the report that homogenized eggs have a pH of 6; why would embryos be able to withstand a pH of 6 inside eggs while nauplii cannot withstand the same pH in water? A thoroughly documented setup with more life stages and pH measurements using microelectrodes directly on different life stages could confirm these observations and potentially explain how eggs are able to withstand conditions that nauplii, copepodites and adult animals reportedly cannot.

The high mean survival rate of resting eggs in hypoxia after 4 months of arrest may seem remarkably different from the findings in previous studies e.g. in [30], Fig. 4B where the number of hatchable eggs decrease substantially after 3 months. After careful inspection of the experimental setups and data presentations in previous studies, we in fact find no such conflicts as the observed differences seem to arise from differing setup strategies. For example, the experimental setup temperature in a recent paper [37] is much higher than it would be in realistic winter conditions as the study focus was different from the present one. Moreover, several studies use hatching success instead of survival rate as an indicator of resting egg performance [20, 30, 37]. Crucially, the hatching success metric fails to account for egg loss: any egg that is not structurally intact at the time of measurement will not be subtracted from the hatching success. Hence, the relative importance of each hatching egg will increase over time, since the number of eggs decrease drastically. As the data in e.g. Fig. 4 in [30] and Fig. 2a of this study show, the egg loss during long term experiments can range from 0 to 90%, depending on conditions, whereas the egg loss is here shown to be negligible in hypoxic conditions. In other words, while hatching success is a valid parameter to describe egg banks, it is an inadequate metric for inferences about the resting abilities of eggs. In another recent paper [29], eggs were taken from a presumably hypoxic sediment and stored in normoxic conditions for 2 months before the experiment was conducted. At this point, after 2 months of normoxic conditions, the authors found a hatchability of the eggs of 97 ± 3 s.d. out of 150 eggs. This supports the present finding that 50–60% of eggs survive 2 months of normoxic resting (Fig. 2b). Presumably, the increased metabolism, which resulted from high Oxygen levels, had depleted the egg resources at this point, which is why the data in [29] does not show a clear difference in hatching between normoxic and hypoxic resting. Furthermore, the experimental handling could compound the rapid decline in the number of viable eggs over time. Interestingly, the data does show a remarkable difference in hatching rate between embryonic arrest at 5 °C and lower temperatures, with a much higher survival rate of eggs at colder temperatures, possibly a result of the lower metabolic rate expected in lower temperatures.

Conversely, an elegant study conducted on a closely-related species found resting *Acartia bifilosa* eggs to have a high survival rate over several months in anoxia, though aerated controls were not provided to document the importance of Oxygen [39]. In this way, the findings on *A. bifilosa* support the findings here. Embryonic development at low temperatures for *A. tonsa* was demonstrated by Hansen et al. [26], and explains the likelihood of a trigger mechanism different from temperature to direct the entry to and exit from embryonic arrest, though the egg development at low temperatures is disputed [45]. Strong winds in the autumn are thought to produce enough turbulence in the seabed to distribute copepod eggs between the benthic hypoxic zone and the largely normoxic zone on top of the seabed. Strong currents and storm events in the spring brings sediment particles many times larger than copepod eggs into suspension [46]. Potentially, this simultaneously bring eggs from the hypoxic zone into the normoxic zone and hereby seed the pelagic waters with copepods [47]. Further, the Oxygen availability in the seabed likely changes over the course of the winter, exposing many copepod eggs to varying conditions. The time frame of resting egg survival found in this study as well as the simplified experimental setup does not entirely explain the ability of *A. tonsa* to appear after the cold winters, when the adult *A. tonsa* population in coastal waters are miniscule [34]. Thus, we suggest to further investigate the resting capabilities of *A. tonsa* eggs covering realistic winter like time frames and examining the potential abilities of eggs produced in water with autumn like temperature, potentially triggering the production of other types of resting eggs [45]. Together, our results do open the possibility that the *A. tonsa* animals which appear in the boreal spring, can be the result of the ability of eggs to undergo a near lossless arrest induced by Oxygen limitation and low temperature. The eggs in this study are produced at 17 °C and thus predominately subitaneous eggs. They are then forced into quiescence during storage at 5 °C. While this scenario is not thought to be dominating in nature, it is realistic in systems with a pycnocline: *A. tonsa* eggs deposited in warm surface water (e.g. 17 °C) will sediment into much colder water (e.g. 5 °C), and, depending on temperature, light, and oxygen conditions, progress through life stages or enter quiescence [48].

## Methods

The *A. tonsa* culture used for all experiments was obtained in 1981 from individuals isolated off the coast of Helsingør, in Øresund, Denmark [16] and assigned the code DFU-ATI. The culture was started at the Danish Institute for Fisheries and Marine Research and has been continuously maintained for > 35 years without being restocked with wild animals. The physiology and ecology of the culture has been described in several publications [15, 16, 20, 29, 30, 41, 43].

The *A. tonsa* culture was maintained in 0.2 µm filtered seawater collected near the site where the culture originated. The culture was kept in 70 L plastic buckets in a sTable 17 °C environment and fed an excess of the microalga *Rhodomonas salina* according to Berggreen et al. [25]. The salinity of the seawater was measured several times during the experiments to be 32 ppt ± 1 s.d. Eggs were harvested daily by siphoning them from the bottom of the buckets and copepods in the pelagic life stages were removed by sequential filtering (250 µm, 125 µm, and 70 µm, respectively). Thus, all eggs used in this study were at most 24 h old at the time of harvest.

The harvested eggs were flushed with filtered seawater to remove faecal pellets, decomposing algae, copepods and other debris that had collected from the bottom of the culture tanks and stuck to the spines of the eggs [23]. The cleaning of the eggs was confirmed visually using a dissecting microscope. Each of the six experiments in this study was set up independently. Exp. 1 confirms the Oxygen permeability of polypropylene tubes and the impermeability of air tight gas vials. Exp. 2–5 was set up with eggs from the long term *A. tonsa* culture and each had a different aim: Exp. 2 explores how the number of eggs per mL affects the pH and survival after a set amount of time (50 days). Exp. 3 explores the long term fate of eggs experiencing hypoxic conditions, Exp. 4 is a replicate setup of Exp. 3, and Exp. 5 explores the long term fate of eggs experiencing saturated levels of Oxygen. For Exp. 3–5, we identified the fractions of eggs which vanish, hatch and remain unhatched after several months of storage. Table 1 presents an overview of the six experimental setups and Additional file 1 contains all raw data and calculations used for figures. A cut tip 1 mL pipette was used to sample eggs. All samples were kept at a temperature of 5 °C in a refrigerator during arrest incubations of 0–281 days. To prevent egg mortality/development due to photo-stress, samples were kept in the dark during arrest incubations in all experiments. O_2_ and pH levels were measured after sample acclimatization to room temperature. pH was measured on a Medorex S7 (Nörten-Hardenberg, Germany) connected to a PHM210 from Radiometer (Copenhagen, Denmark), and calibrated between uses. Oxygen concentration was measured using a Unisense Oxygen electrode (OX-200 from Unisense, Aarhus, Denmark) connected to a Picoammeter (P2000 from Unisense, Aarhus, Denmark) and calibrated to 100% and 0% Oxygen concentration by bubbling with atmospheric air and Nitrogen gas, respectively, at each time point. Eggs were set up for hatching by emptying an entire sample container into a 60 mm Petri dish and flushing the container several times with oxygenated 0.2 µm filtered seawater in a total volume of at least 10 mL. Periodic inspections under a dissection microscope showed that all eggs had been removed from the sample containers. For all hatching experiments, Petri dishes with eggs and oxygenated filtered seawater were incubated for 72 h at 17 °C in a climate controlled room with constant light for optimal hatching conditions [49]. After incubation, the samples were fixed in a 1% acid Lugols solution. For Exp. 2, 3, and 4 (Table 1) unhatched eggs and nauplii were counted under a dissecting microscope (Olympus SZ 40, Olympus Optical (Europe) GmBH, Hamburg, Germany). For Exp. 5 (Table 1), eggs were counted before hatching and only nauplii were counted after hatching. For egg loss data, the total number of eggs and nauplii for each time point was compared to the ZTM number of eggs. For survival data, the number of hatched nauplii in each replicate was compared to the ZTM number of eggs (timepoint 0).

For Oxygen permeability measurements and survival data, 0.2 mL polypropylene tubes (Exp. 1 and 5 in Table 1, Microamp Reaction tube with Cap, Applied Biosystems, Foster City, California, United States) and 3 mL or 12 mL air tight glass vials (Exp. 2, 3, and 4 in Table 1, Exetainer, Labco, Lampeter, UK) were filled with 0.2 µm filtered seawater which was Oxygen depleted by Nitrogen gas flushing. Anoxic seawater was added to the maximum volume of the container and the cap was tightened with minimal air bubbles caught under the lid. The 0.2 mL containers used in Exp. 1 were flushed with anoxic seawater with a 1 mL pipette while they were submerged in 0.2 µm filtered seawater to remove any remaining air bubbles. The seawater was kept anoxic by continuous Nitrogen flushing.

To compare the hatching success of eggs which were exposed to Oxygen and which were not exposed to Oxygen, linear regressions were fitted to the hatching success as a function of time with data points < 200 days included. To test whether they were significantly different from each other, an F-test for difference between two regression coefficients was used [50].

## Conclusions

This laboratory study maps the pH and Oxygen availability tolerance of *A. tonsa* eggs in prolonged embryonic arrest. We found a high tolerance of resting eggs to sediment-realistic low pH and documented the necessity of low Oxygen levels for the long term survival of resting eggs. These results can have a profound impact on the understanding of boreal coastal water ecosystems, as they demonstrate that the eggs of an important and globally distributed copepod species in induced arrest are able to tolerate month long arrest with a negligible rate of loss. Previously, the vast majority of eggs in induced arrest were thought to vanish during the winter. Thus, the results of the present study add to the understanding of the pelagic *A. tonsa* annual biomass fluctuations by documenting the importance of oxygen in resting eggs.

## Additional file

**Additional file 1.** Raw data and calculations for all experiments as well as an overview of experiments in this study.

## Abbreviations

DO: dissolved oxygen
ZTM: zero time mean
